# The rapid diagnosis and effective inhibition of coronavirus using spike antibody attached gold nanoparticles†

**DOI:** 10.1039/d0na01007c

**Published:** 2021-01-18

**Authors:** Avijit Pramanik, Ye Gao, Shamily Patibandla, Dipanwita Mitra, Martin G. McCandless, Lauren A. Fassero, Kalein Gates, Ritesh Tandon, Paresh Chandra Ray

**Affiliations:** Department of Chemistry and Biochemistry, Jackson State University Jackson MS 39217 USA paresh.c.ray@jsums.edu; Department: Microbiology and Immunology, University of Mississippi Medical Center Jackson MS 39216 USA

## Abstract

Severe acute respiratory syndrome coronavirus 2 (SARS-CoV-2), the cause of the coronavirus disease that began in 2019 (COVID-19), has been responsible for 1.4 million deaths worldwide as of 13 November 2020. Because at the time of writing no vaccine is yet available, a rapid diagnostic assay is very urgently needed. Herein, we present the development of anti-spike antibody attached gold nanoparticles for the rapid diagnosis of specific COVID-19 viral antigen or virus *via* a simple colorimetric change observation within a 5 minute time period. For rapid and highly sensitive identification, surface enhanced Raman spectroscopy (SERS) was employed using 4-aminothiophenol as a reporter molecule, which is attached to the gold nanoparticle *via* an Au–S bond. In the presence of COVID-19 antigen or virus particles, owing to the antigen–antibody interaction, the gold nanoparticles undergo aggregation, changing color from pink to blue, which allows for the determination of the presence of antigen or virus very rapidly by the naked eye, even at concentrations of 1 nanogram (ng) per mL for COVID-19 antigen and 1000 virus particles per mL for SARS-CoV-2 spike protein pseudotyped baculovirus. Importantly, the aggregated gold nanoparticles form “hot spots” to provide very strong SERS signal enhancement from anti-spike antibody and 4-aminothiophenol attached gold nanoparticles *via* light–matter interactions. Finite-difference time-domain (FDTD) simulation data indicate a 4-orders-of-magnitude Raman enhancement in “hot spot” positions when gold nanoparticles form aggregates. Using a portable Raman analyzer, our reported data demonstrate that our antibody and 4-aminothiophenol attached gold nanoparticle-based SERS probe has the capability to detect COVID-19 antigen even at a concentration of 4 picograms (pg) per mL and virus at a concentration of 18 virus particles per mL within a 5 minute time period. Using HEK293T cells, which express angiotensin-converting enzyme 2 (ACE2), by which SARS-CoV-2 enters human cells, we show that anti-spike antibody attached gold nanoparticles have the capability to inhibit infection by the virus. Our reported data show that antibody attached gold nanoparticles bind to SARS-CoV-2 spike protein, thereby inhibiting the virus from binding to cell receptors, which stops virus infection and spread. It also has the capability to destroy the lipid membrane of the virus.

## Abstract 2

Severe acute respiratory syndrome coronavirus 2 (SARS-CoV-2), the cause of the coronavirus disease that began in 2019 (COVID-19), has been responsible for 1.4 million deaths worldwide as of 13 November 2020.

## Graphical abstract

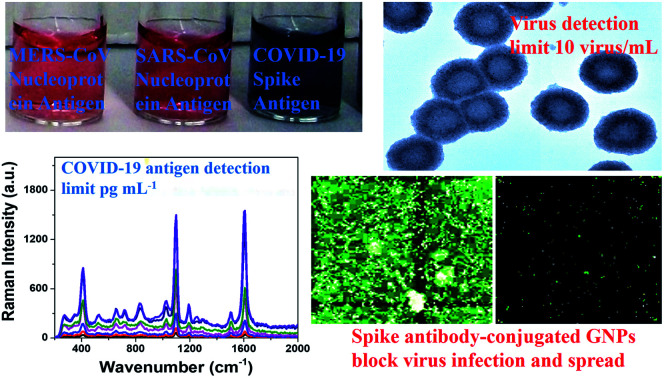

## Introduction

The outbreak of severe acute respiratory syndrome coronavirus-2 (SARS-CoV-2), also known as coronavirus disease of 2019 (COVID-19), is responsible for huge global disruption to health and economies.^1–10^ As of 13 November 2020, more than 59.4 million people have suffered from COVID-19 disease and around 1.40 million have died worldwide^1,2^. It is now well documented that spike (S), envelope (E), membrane (M), and nucleocapsid (N) are the important structural proteins for coronavirus particles.^6–16^ Several reports indicate that spike protein plays the most important role for virus entry and disease pathogenesis.^3–14^ Because at the time of writing there are no FDA-approved vaccines or therapeutics for the treatment of COVID-19 disease, rapid diagnosis is the key to slowing the spread of COVID-19 and saving lives.^1–20^ Driven by the urgent need, we report the development of an anti-spike antibody attached gold nanoparticle (GNP) for rapid diagnosis of specific COVID-19 viral antigen (COVID-19 Spike Recombinant Antigen) or the virus itself *via* naked eye colorimetric change and highly sensitive surface enhanced Raman spectroscopy (SERS). Recently, Ventura *et al.* reported highly sensitive COVID-19 detection by means of a colorimetric assay that employed GNPs to which three different (spike, envelope, and membrane) antibodies were attached.^14^ In the current manuscript, we show that anti-spike antibody attached GNPs are capable of ultrasensitive identification as well as inhibition of virus particles.

As we and others have reported before, owing to good biocompatibility, size dependent optical properties and ease of modification of the surface, gold nanoparticles can be used for the development of excellent biosensing platforms.^17–33^ As shown in Fig. S1 and S2 in the ESI,† the color of our anti-spike antibody attached gold nanoparticle is pink and it changed to a bluish tinge in the presence of the COVID-19 antigen or virus owing to aggregation *via* antigen–antibody interaction. As a result, we observed a color change upon aggregation. Based on color observation with the naked eye, we can detect COVID-19 antigen even at a concentration of 1 nanogram (ng) per mL and virus particles at a concentration of 1000 virus particles per mL. Since SARS-CoV-2 is a biosafety-level-3 (BSL-3) virus, we have used a BSL-2 SARS-CoV-2 spike protein pseudotyped baculovirus (pseudo SARS-CoV-2) as a model system for our diagnosis and inhibition experiments.^36,37^

Owing to its extremely high sensitivity, SERS can be used for detection of very low levels of viruses, bacteria, cancer cells and different biomolecules.^17–27^ It is now well documented that Raman signals can be enhanced by several orders (10^7^ to 10^10^) of magnitude by using plasmonic nanoparticles *via* the electromagnetic mechanism (EM) and chemical mechanism (CM).^17–27^ For rapid and highly sensitive identification, SERS has been employed using 4-aminothiophenol (4-ATP) as reporter molecule, attached to gold nanoparticles *via* Au–S bonds. In the presence of COVID-19 antigen or virus particles, the aggregated nanoparticles form “hot spots”. As we and other groups have reported, that “hot spot” formation is a very important factor to provide extraordinary enhancement in SERS;^17–27^ in the presence of COVID-19 antigen or virus particles strong SERS signals from 4-aminothiophenol have been observed, which allow diagnosis of viral antigen even at a concentration of 4 picograms (pg) per mL and virus at 18 virus particles per mL concentration.

It is now well documented that for SARS-CoV-2, the viral infection starts with the binding of viral particles to receptors on the host cells, mediated by the virus spike protein.^8–17,34–41^ As different studies indicate that spike protein binds specifically to the human angiotensin-converting enzyme 2 (ACE2) receptor on the surface of human cells,^8–17^ scientists from several groups are developing inhibitors that have the capability to block the mechanism by which the virus binds to the ACE2 receptor.^34–41^ Herein, using pseudo SARS-CoV-2 as the model virus, we demonstrate that anti-spike antibody attached gold nanoparticles have the capability to block viral entry into cells. Pseudo SARS-CoV-2 is a BSL-1 baculovirus pseudotyped with spike (S) protein. Using HEK293T cells, which express ACE2, by means of which SARS-CoV-2 enters human cells, we demonstrate that anti-spike antibody attached gold nanoparticles block viral replication and virus spread in HEK293T cells.

## Experimental section

### Citrate coated gold nanoparticle synthesis

**Fig. 1:**
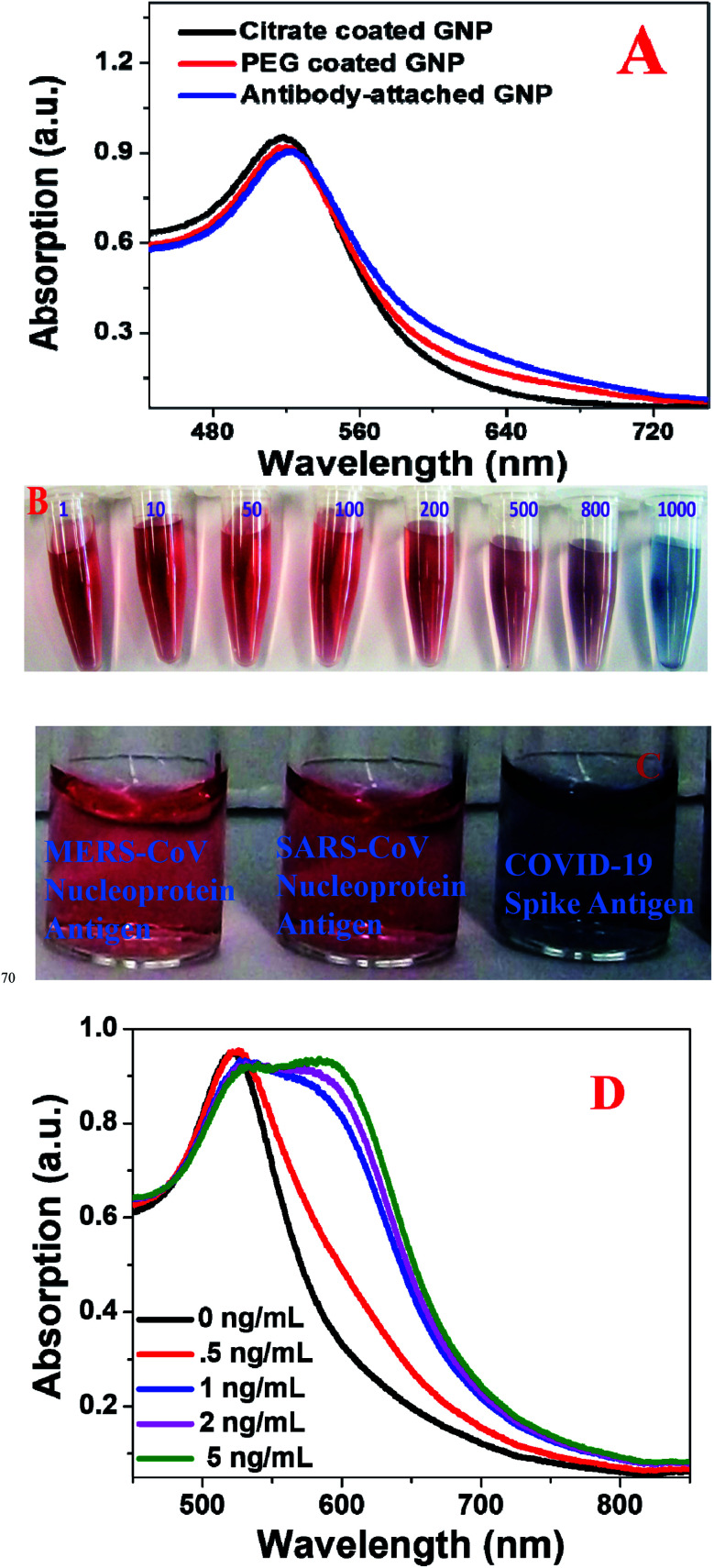
(A) Absorption spectra from citrate coated gold nanoparticles, PEG coated gold nanoparticles, and anti-spike antibody attached gold nanoparticles. (B) The color of antibody attached GNPs in the presence of different amounts of COVID-19 antigen from 1 pg mL^−1^ to 1000 pg mL^−1^. (C) Selectivity of the anti-spike antibody attached gold nanoparticle-based colorimetric assay for COVID-19 antigen (5 ng mL^−1^). No color change was observed in the presence of 5 ng mL^−1^ MERS-CoV nucleoprotein antigen and 5 ng mL^−1^ SERS-CoV nucleoprotein antigen. (D) Absorption spectra from antibody attached GNPs in the presence of different concentrations of COVID-19 antigen.

As shown in Fig. S1A in the ESI,† citrate coated gold nanoparticles (GNPs) were synthesized using HAuCl_4_·3H_2_O and sodium citrate, as we and others have reported before.^17–30^ For this purpose, we used a 0.01% solution of HAuCl_4_·3H_2_O and 1 wt% of sodium citrate. Transmission electron microscopy (TEM), and colorimetric and dynamic light scattering (DLS) data were used to characterize the freshly synthesized nanoparticles. Table 1 shows the DLS data, indicating that the size of the freshly prepared citrate coated GNPs is ∼15 ± 2 nm. The inset picture in Fig. S1A† shows the color of freshly prepared GNPs is pink. As we and others have reported before, GNPs exhibit unique optical properties due to surface plasmon resonance (SPR).^17–30^ It is well documented that the SPR band for citrate coated gold nanoparticles is due to phase changes resulting from the increased rate of electron-surface collisions compared with larger particles.^28–33^Fig. 1A shows the absorption spectra of citrate coated gold nanoparticles, in which *λ*_max_ is seen to be ∼520 nm. As we and others have reported before,^20–33^ SPR of gold nanoparticles yields exceptionally high absorption, with extinction coefficient *ε*_(15)520nm_ = 3.6 × 10^8^ cm^−1^ M^−1^, which we used here for sensing of COVID-19 antigen or virus using naked eye colorimetric study.

**Table 1:** Size of GNPs under different coating conditions, measured *via* dynamic light scattering. This table also reports how the size of anti-spike antibody attached GNPs varies in the presence of different amounts of COVID-19 antigen *via* aggregation

System	Size measured by DLS
Citrate coated GNPs	15 ± 2 nm
PEG coated GNPs	18 ± 4 nm
Antibody coated GNPs	27 ± 6 nm
Antibody attached GNPs (1.3 nM) with 1 pg mL^−1^ antigen	60 ± 30 nm
Antibody attached GNPs (1.3 nM) with 100 pg mL^−1^ antigen	120 ± 60 nm
Antibody attached GNPs (1.3 nM) with 500 pg mL^−1^ antigen	200 ± 80 nm
Antibody attached GNPs (1.3 nM) with 1 ng mL^−1^ antigen	700 ± 300 nm
Antibody attached GNPs (1.3 nM) with 5 ng mL^−1^ antigen	900 ± 300 nm

The X-ray diffraction (XRD) spectrum from citrate coated gold nanoparticles shown in Fig. S2D† shows (111), (200), (220) and (311) diffraction peaks, due to face centered cubic gold (JCPDS 04-0784).^20–30^ The particle concentration was measured by UV-visible spectroscopy using the molar extinction coefficient at the wavelength of the maximum absorption of the gold colloid (*ε*_(15)519nm_ = 3.6 × 10^8^ cm^−1^ M^−1^), as we and others have reported before.^28–30^

### PEG coated gold nanoparticle synthesis

In the next step, as shown in Fig. S1B in the ESI,† to render the GNPs biocompatible, they were coated with HS-PEG-COOH. For this purpose, we used carboxy-PEG12-thiol (HS-PEG12-COOH). We dissolved 10 mg of HS-PEG12-COOH in 5 mL of water and then added citrate coated GNPs using a syringe and vigorous stirring. After sonication of the mixture for 30 minutes, the final products were separated through centrifugation. As reported in Table 1, after PEG coating, the size of the GNPs had increased to ∼18 ± 4 nm. The inset picture in Fig. S1B† shows that the color of freshly prepared PEG coated GNPs is pink. Fig. 1A shows the absorption spectrum of PEG coated gold nanoparticles, in which *λ*_max_ is ∼521 nm, which indicates that after coating with PEG, the optical spectrum for GNPs remains almost the same.

### Anti-spike antibody coated gold nanoparticle synthesis

For targeted diagnosis and inhibition, we developed anti-spike antibody attached gold nanoparticles. For this purpose, we used EDC (1-ethyl-3-(3-dimethylaminopropyl)-carbodiimide)/NHS (*N*-hydroxysuccinimide) chemistry. In short, we added 0.2 molar (M) EDC and 0.05 M *N*-hydroxysulfosuccinimide sodium salt [1 : 3 (v/v) ratio] to a solution containing GNPs and anti-spike antibody, and the mixture was sonicated for 30 minutes. After that, anti-spike antibody attached GNPs were separated from GNPs without antibody *via* centrifugation for 15 minutes at 6000 rpm, followed by resuspension in buffer. As reported in Table 1, after antibody attachment the size of GNPs had increased to ∼27 ± 6 nm. To determine how many antibodies were attached to the GNPs, we synthesized Cy3 dye attached anti-spike antibody conjugated GNPs. After separation of the Cy3 dye attached antibody conjugated GNPs from unconjugated molecules, we added 10 μM potassium cyanide to oxidize the GNPs. After that, from fluorescence data we estimated that about 10 ng mL^−1^ antibodies were attached on GNPs.

**Fig. 2:**
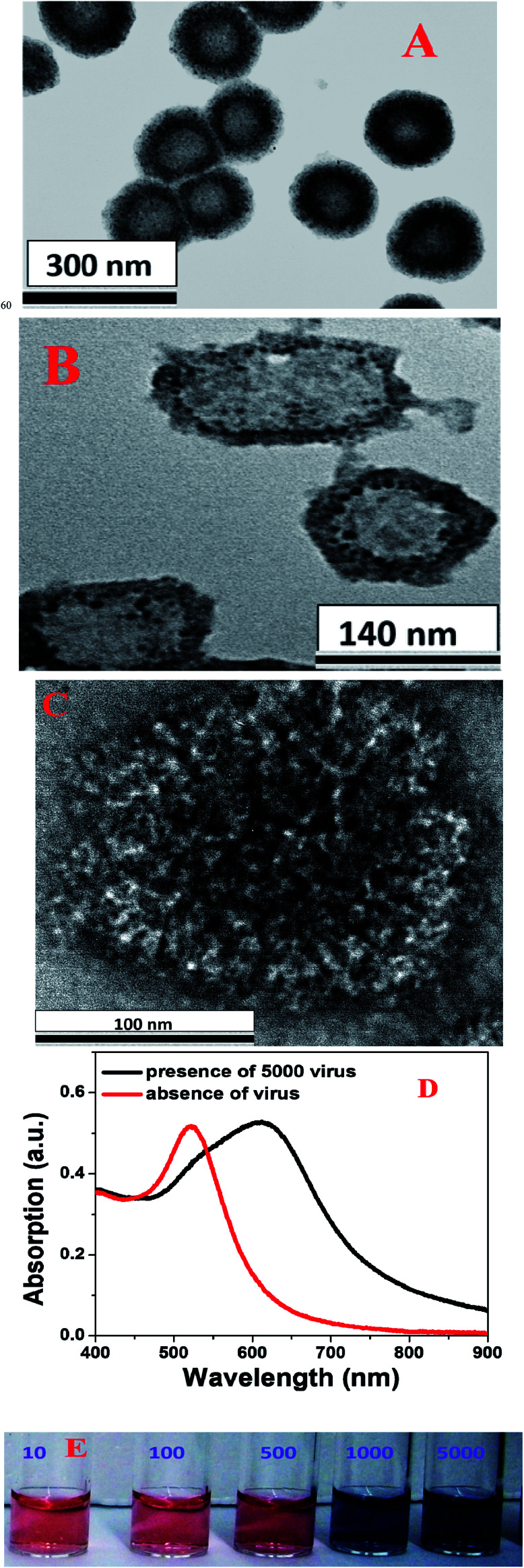
(A) A TEM image showing the morphology of pseudo SARS-CoV-2, which we used for virus diagnosis. (B) A TEM image showing the morphology of antibody attached GNP conjugated pseudo SARS-CoV-2 when we used 20 pM gold nanoparticles. (C) A TEM image showing the morphology of anti-spike antibody attached gold nanoparticle conjugated pseudo SARS-CoV-2 when we used 800 pM gold nanoparticles. (D) Variations in the absorption spectrum of antibody attached GNPs in the presence and absence of pseudo SARS-CoV-2. (E) Changes in the color of antibody attached GNPs in the presence of different amounts of pseudo SARS-CoV-2 (10–5000 virus particles per mL).

The Fourier-transform infrared (FTIR) spectrum from anti-spike antibody attached gold nanoparticles shown in Fig. S2E† contains amide-A, amide-I, amide-II and amide-II bands, which indicates that anti-spike-antibodies are attached on the surface of the gold nanoparticles. The inset picture in Fig. S1C† shows the color of freshly prepared antibody GNPs is pink. Fig. 2D shows the absorption spectra of antibody attached gold nanoparticles, in which *λ*_max_ is ∼522 nm, which indicate that after attaching antibody, the optical spectrum for GNPs remains almost the same.

The X-ray diffraction (XRD) spectra from antibody attached gold nanoparticles as reported in Fig. S2D† show (111), (200), (220) and (311) diffraction peaks, which indicate that the crystal structure remains the same after conjugation with antibody.

### Surface-enhanced Raman spectroscopy using anti-spike antibody attached gold nanoparticles

For the detection of COVID-19 antigen and virus using SERS, we used our developed portable Raman spectrometer with laser excitation of 670 nm.^22–24,26,27^ In our design we have used an InPhotonics 670 nm Raman fiber optic probe for excitation and data collection. For SERS signal collection, we used a miniaturized QE65000 Scientific-grade Spectrometer from Ocean Optics.^22–24,26,27^ All SERS spectra were taken on drop-cast dried glass slides. For Raman data collection we used 10 second acquisition time and 5-scan averaging, so that we could achieve very good signal-to-noise ratio.^22–24,26,27^

### SARS-CoV-2 pseudotype particles

Pseudo SARS-CoV-2 (Cat. # C1110G) was purchased from Montana Molecular, Bozeman, MT 59715, USA. HEK293T cells (ATCC # CRL3216) plated in twelve-well tissue culture dishes were infected with the pseudotyped virus with a dilution range of 10^2^ to 10^7^, and virus titers were calculated by counting green fluorescent protein (GFP) positive cells under a fluorescence microscope.^36–38^

### Virus inhibition assays

HEK293T cells (ATCC # CRL3216) were plated on a 96-well plate in complete medium (DMEM + 10% FBS) and incubated under normal growth conditions (5% CO_2_ and 37 °C, protected from light) for 12–24 hours.^36–38^ These HEK293T cells express low levels of ACE2 receptor which are sufficient for SARS-CoV-2 entry. Dilutions of test nanoparticles were made in DMEM with a final volume of 100 μL each.^36–38^ The pseudovirus stock (2.5 μL of the 2 × 10^10^ units permL stock) was mixed with the diluted nanoparticles and incubated for 1 h at 37 °C, then laid over HEK293T cells plated in the 96-well tissue culture dishes, along with 0.6 μL of 500 nM sodium butyrate (to give a final concentration of 2 mM). Plates were incubated at 37 °C under 5% CO_2_ for 48 h. Cells were fixed in 3.7% formaldehyde and the assay was read on a Cytation 5 automated fluorescence microscope (BioTek Instruments, Inc., Winooski, VT, USA).^36–38^

## Results and discussion

### Naked eye colorimetric diagnosis of COVID-19 antigen using anti-spike antibody attached gold nanoparticles

Antigen tests are used in clinics to diagnose different virus infections, and now the FDA has approved rapid antigen tests that can identify SARS-CoV-2.^2^ Here, our experimental data indicate that naked eye colorimetric diagnosis of COVID-19 antigens can be performed within 5 minutes using anti-spike antibody attached gold nanoparticles. As shown in Fig. S1D in the ESI,† naked eye colorimetric diagnosis of COVID-19 antigens using antibody attached GNPs is based on the fact that in the presence of COVID-19 antigen, as a result of antigen–antibody interaction, antibody attached GNPs aggregate, as shown in Fig. S2A–C in the ESI.† As a result, as shown in Fig. 1A–C, we have observed the color of the suspension of antibody attached GNPs change from pink to blue, which is due to the surface plasmon coupling between antibody attached GNPs. The TEM images in Fig. S2A–C† and DLS data in Table 1 show that as we increased the amount of COVID-19 antigen, the size of anti-spike antibody attached gold nanoparticles increased tremendously owing to aggregation. As we and others have reported before, when the interparticle distances are ∼2.5 times lower than the diameter of the nanoparticles, the aggregation of gold nanoparticles will induce the coupling of the plasmon modes very strongly, which will induce a red shift of the absorption spectrum.^28–33^ The absorption spectra shown in Fig. 1D show a red shift from 520 to 600 nm for aggregated antibody attached GNPs. The reported absorption spectra also indicate the broadening of the plasmon resonance peak after aggregation of antibody attached GNPs, which could be due to longitudinal resonance.

To determine the sensitivity of naked eye colorimetric assay for the identification of COVID-19 antigen using antibody attached GNPs, we performed an antigen concentration dependence study using from 1 pg mL^−1^ to 10 000 pg mL^−1^ COVID-19 antigen. As shown in Fig. 1B, naked eye colorimetric assay can be used for the diagnosis of a minimum concentration of 1000 pg mL^−1^ or 1 ng mL^−1^ antigen. Next, we determined the selectivity of the naked eye colorimetric assay for the identification of COVID-19 antigen using antibody attached GNPs. Since COVID-19, severe acute respiratory syndrome coronavirus (SARS-CoV) and Middle East respiratory syndrome coronavirus (MERS-CoV) are all coronaviruses, we also performed the same experiment for SARS-CoV virus antigen and MERS-CoV antigen separately using anti-spike antibody attached gold nanoparticles. As shown in Fig. 1C, we did not observe any colorimetric change even when 5 ng mL^−1^ SARS-CoV virus antigen or MERS-CoV antigen was added to the pink solution of anti-spike antibody attached gold nanoparticles. The above reported data clearly show that the antibody attached GNP-based naked eye colorimetric assay is highly selective for COVID-19 spike-antigen.

### Naked eye colorimetric diagnosis of pseudo SARS-CoV-2 using antibody attached GNPs

As we have discussed above, since SARS-CoV-2 is a biosafety-level-3 (BSL-3) virus, we used a BSL-2 pseudo SARS-CoV-2 with spike (S) protein as a model system for our diagnosis experiment.^36,37^ As shown in Fig. S1E and 2E,† naked eye colorimetric diagnosis of pseudo SARS-CoV-2 using antibody attached GNPs is based on the fact that in the presence of pseudo SARS-CoV-2, as a result of the virus spike protein–antibody interaction, antibody attached GNPs aggregate on the surface of the virus, as shown in Fig. 2A–C. Since the size of the pseudo SARS-CoV-2 is around 120–160 nm, several antibody attached GNPs can aggregate on the surface of each virus particle. The TEM image in Fig. 2C shows that as we increased the concentration of antibody attached GNPs, owing to the virus spike protein–antibody interaction, antibody attached GNPs aggregated and covered the entire surface of the virus particles. As a result, as shown in Fig. 2E, we observed the color of the suspension of antibody attached GNPs change from pink to blue, as a result of surface plasmon coupling between antibody attached GNPs. Our reported naked eye colorimetric diagnosis of pseudo SARS-CoV-2 using antibody attached GNPs is very quick and it takes less than 5 minutes to get the results. The absorption spectra in Fig. 2D show a red shift of absorption from 520 to 640 nm for aggregated antibody attached GNPs in the presence of pseudo SARS-CoV-2.

To determine the sensitivity of naked eye colorimetric assay for the identification of pseudo SARS-CoV-2 using antibody attached GNPs, we performed a pseudo SARS-CoV-2 concentration dependence study from 10 virus particles per mL to 5000 virus particles per mL. As shown in Fig. 2E, naked eye colorimetric assay can be used for the diagnosis of a minimum concentration of 1000 virus particles per mL of pseudo SARS-CoV-2. Our reported naked eye colorimetric assay for SARS virus detection exhibits slightly higher sensitivity than the antibody attached GNP-based influenza A virus detection as reported by Liu *et al.*^40^

### Highly sensitive SERS diagnosis of COVID-19 antigen and pseudo SARS-CoV-2 using antibody attached GNPs

**Fig. 3:**
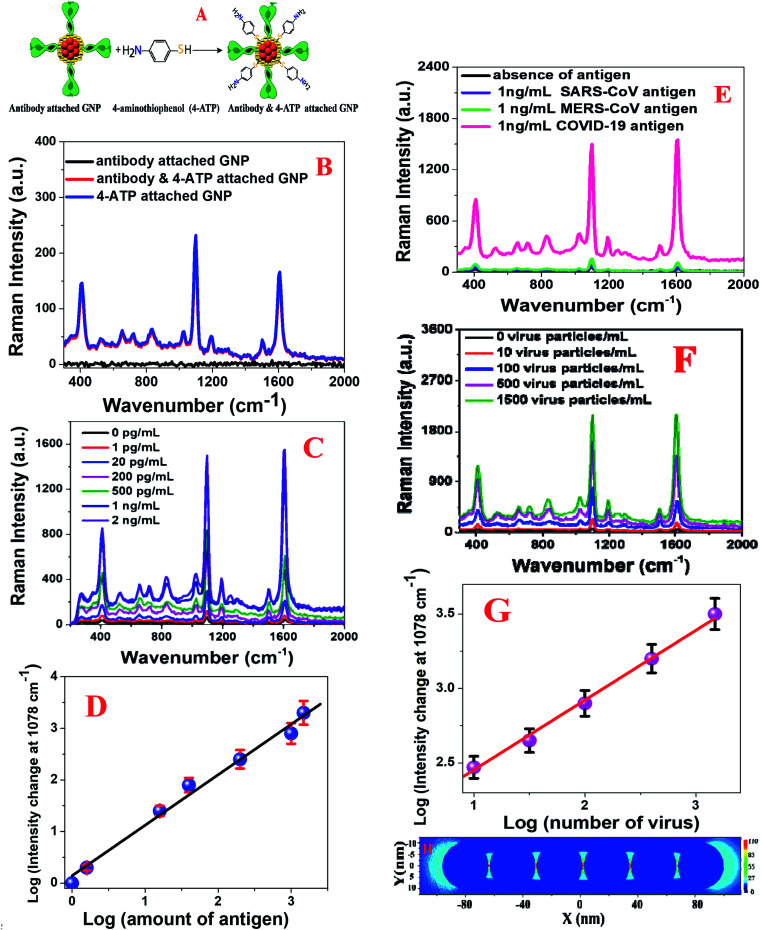
(A) A schematic diagram showing the synthetic path we used for the development of antibody and 4-aminothiophenol coated gold nanoparticles. (B) SERS intensity from anti-spike antibody (10 ng mL^−1^) attached gold nanoparticles (1.3 nM), 4-aminothiophenol (300 nM) attached gold nanoparticles (1.3 nM), and anti-spike antibody (10 ng mL^−1^) as well as 4-aminothiophenol (300 nM) attached gold nanoparticles (1.3 nM). (C) Variations in the SERS intensity from anti-spike antibody and 4-aminothiophenol attached gold nanoparticles in the presence of different concentrations of COVID-19 antigen (COVID-19 Spike Recombinant Antigen). (D) Variations in the Raman intensity change at 1078 cm^−1^ from anti-spike antibody and 4-aminothiophenol attached gold nanoparticles with the concentration of COVID-19 Antigen (0 pg mL^−1^ to 2 ng mL^−1^). (E) Variations in the SERS intensity from anti-spike antibody and 4-aminothiophenol attached gold nanoparticles in the presence of different virus antigens. (F) Variations in the SERS intensity from the anti-spike antibody and 4-aminothiophenol attached gold nanoparticles in the presence of different amounts of pseudo SARS-CoV-2 (number of virus particles per mL). (G) Variations in the Raman intensity change at 1078 cm^−1^ from anti-spike antibody and 4-aminothiophenol attached gold nanoparticles with the concentration of pseudo SARS-CoV-2 (number of virus particles per mL). (H) FDTD simulation data showing how the (|*E*|^2^) profile varies with an increase in the number of gold nanoparticles in aggregates.

Although naked eye colorimetric assay is capable of the identification of COVID-19 antigen and pseudo SARS-CoV-2 within a 5 minute time period, the sensitivity is low. To enhance the sensitivity significantly, we employed SERS for the diagnosis of COVID-19 antigen and pseudo SARS-CoV-2. As shown in Fig. 3A, for rapid and highly sensitive identification *via* SERS, we used 4-aminothiophenol as reporter molecule, which is attached to gold nanoparticles *via* Au–S bonds. Synthesis details are reported in the ESI.†

Fig. 3B shows the SERS spectra from anti-spike antibody (10 ng mL^−1^) only attached GNPs (1.3 nM), 4-aminothiophenol (300 nM) only attached GNPs (1.3 nM) and antibody (10 ng mL^−1^) as well as 4-aminothiophenol (300 nM) attached gold nanoparticles (1.3 nM). As shown in Fig. 3B, in the absence of the 4-aminothiophenol Raman reporter, we did not see any Raman signal, which clearly shows that the observed Raman spectrum is mainly due to the presence of the 4-aminothiophenol Raman reporter. The Raman spectrum in Fig. 3B from anti-spike antibody and 4-aminothiophenol attached gold nanoparticles shows Raman peaks due to the a_1_ vibrational modes: *ν*(C–C + NH_2_ bend) at ∼1590 cm^−1^ and *ν*[*ν*(C–C) + *δ*(C–H)] at ∼1078 cm^−1^.^17–27^ Similarly, we also observed b_2_ mode Raman peaks from the anti-spike antibody and 4-aminothiophenol attached gold nanoparticles, as shown in Fig. 3B: CC stretch in Ph ring + NH_2_ rock at ∼1435 cm^−1^ and *ν*(C–N) + *ν*(C–S) + *γ*(CCC) vibrational modes at 464 cm^−1^.^17–27^

Our SERS-based diagnosis of COVID-19 antigens using antibody attached GNPs is based on the fact that in the presence of COVID-19 antigen, owing to the antigen–antibody interaction, antibody attached GNPs aggregate and form several “hot spots”, as shown in Fig. S2A–C in the ESI.† It is now well documented that “hot spot” formation is a very important factor to provide extraordinary enhancement in SERS, owing to the strong light–matter interaction *via* plasmon-excitation coupling.^17–27^ As shown in Fig. 3C, very strong SERS signals from anti-spike antibody and 4-aminothiophenol attached gold nanoparticles are observed in the presence of COVID-19 Spike Recombinant Antigen; this is due to the formation of huge amounts of electromagnetic “hotspots”, which enhances Raman intensity by several orders of magnitude *via* light–matter and matter–matter interactions.^17–27^

To understand better how “hot spot” formation greatly enhances the SERS signal, we performed finite-difference time-domain (FDTD) simulation.^22–24,42,43^ Theoretical details have been reported before by our group and other groups.^22–24,26,27,42,43^ For the simulation, we used 20 nm spherical gold nanoparticles, 670 nm wavelength, 0.001 nm mesh resolution, and 4000 fs time.^22–24,26,27^Fig. 3H shows how the square of field enhancement (|*E*|^2^) varies with distance for nanoparticles. The FDTD simulation data in Fig. 3H indicate a more than 2 orders of magnitude higher field enhancement in “hot spot” positions when gold nanoparticles formed aggregates. As we and others have reported before, Raman enhancement is proportional to |*E*|^4^,^22–24,26,27,42,43^ and as a result, reported FDTD simulation data indicate that there is a possibility of 4 orders of magnitude Raman enhancement in “hot spot” positions when gold nanoparticles form aggregates.

Having observed a huge Raman signal enhancement in the presence of COVID-19 antigen, to determine the sensitivity of SERS assay for the identification of COVID-19 antigen using anti-spike antibody and 4-aminothiophenol attached gold nanoparticles, we performed an antigen concentration dependence study using 1 pg mL^−1^ to 2000 pg mL^−1^ COVID-19 antigen. As shown in Fig. 3C, SERS assay can be used for the diagnosis of a minimum concentration of 1 pg mL^−1^ antigen, where we observed a more than 2-fold increment of the Raman signal. Fig. 3D shows how the Raman signal at 1078 cm^−1^ from anti-spike antibody and 4-aminothiophenol attached gold nanoparticles varies with the concentration of COVID-19 antigen.

To determine the limit of detection (LOD), we used the following equation:^22–24^

1 LOD = 3*σ*/*S*

where *σ* is the standard deviation of the blank and *S* is the slope of the calibration curve. For the determination of the standard deviation of the blank, we used the baseline noise. Using the concentration–dependence plot shown in Fig. 3D, we estimated the LOD to be ∼4 pg mL^−1^ for SERS assay using COVID-19 antigen. Next, we determined the selectivity of the SERS assay for the identification of COVID-19 antigen using anti-spike antibody and 4-aminothiophenol attached gold nanoparticles. For this purpose, we also performed the same SERS experiment for SARS-CoV virus antigen and MERS-CoV antigen separately using anti-spike antibody and 4-aminothiophenol attached gold nanoparticles. As shown in Fig. 3E, we did not observe any SERS intensity change even when 1 ng mL^−1^ SARS-CoV antigen or MERS-CoV antigen was added to anti-spike antibody and 4-aminothiophenol attached gold nanoparticles. The above reported data clearly show that antibody attached GNP-based SERS is highly selective for COVID-19 spike antigen.

Our SERS-based diagnosis of pseudo SARS-CoV-2 using anti-spike antibody and 4-aminothiophenol attached gold nanoparticles is based on the fact that in the presence of pseudo SARS-CoV-2, owing to the virus spike protein–antibody interaction, anti-spike antibody and 4-aminothiophenol attached gold nanoparticles aggregate on the surface of the virus, as shown in Fig. 2B and C. Since the size of the pseudo SARS-CoV-2 is around 120–150 nm, several anti-spike antibody and 4-aminothiophenol attached gold nanoparticles can aggregate on the surface of each virus particle and form several “hot spots”. As a result, as shown in Fig. 3F, we observed a huge SERS intensity change, which is due to the surface plasmon coupling between anti-spike antibody and 4-aminothiophenol attached gold nanoparticles. Our reported SERS diagnosis of pseudo SARS-CoV-2 using anti-spike antibody and 4-aminothiophenol attached gold nanoparticles is very quick: it takes less than 5 minutes to get the results.

To determine the sensitivity of SERS assay for the identification of pseudo SARS-CoV-2 using anti-spike antibody and 4-aminothiophenol attached gold nanoparticles, we performed a virus concentration dependence study using from 10 to 1500 pseudo SARS-CoV-2 virus particles. As shown in Fig. 3F, SERS assay can be used for the diagnosis of the minimum concentration of 10 virus particles per mL, where we observed a more than 2-fold increment of the Raman signal. Fig. 3G shows how the Raman signal at 1078 cm^−1^ from anti-spike antibody and 4-aminothiophenol attached gold nanoparticles varies with the concentration of pseudo SARS-CoV-2. Using eqn (1) and the concentration-dependent linear plot shown in Fig. 3G, we estimated the LOD to be ∼18 virus particles per mL for SERS assay using pseudo SARS-CoV-2. The sensitivity of our reported SERS detection assay is comparable with the Abbott Real Time SARS-CoV-2 RT-PCR assay (40 copies per mL).^44^

### Inhibition of pseudo SARS-CoV-2 using anti-spike antibody attached gold nanoparticles

**Fig. 4:**
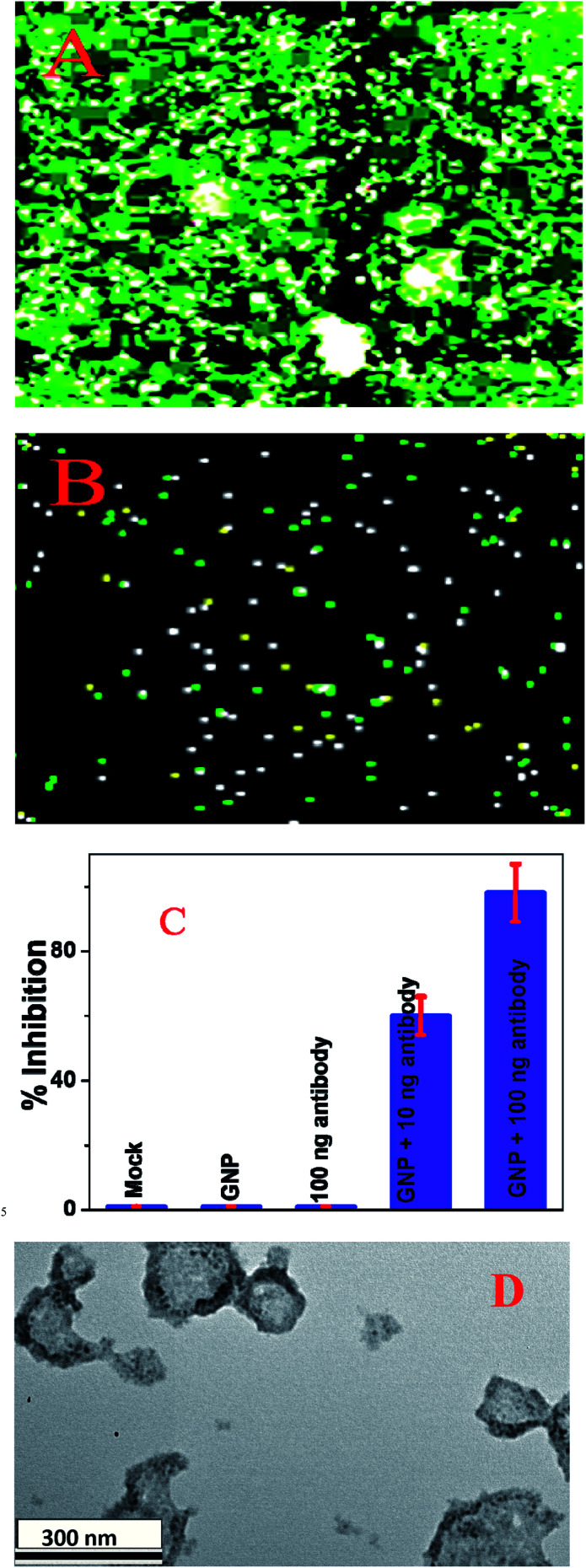
(A) SARS-CoV-2 pseudovirus green fluorescent protein (GFP) expression in infected HEK293T cells in the absence of antibody attached gold nanoparticles. (B) SARS-CoV-2 pseudovirus GFP expression in infected HEK293T cells in the presence of 10 ng mL^−1^ anti-spike antibody. (C) Variations in the inhibition efficiency of SARS-CoV-2 pseudovirus green fluorescent protein (GFP) expression in infected HEK293T cells in the presence of buffer (Mock), gold nanoparticles (GNPs), anti-spike antibody, and anti-spike antibody attached gold nanoparticles. (D) A TEM image showing that 100 ng mL^−1^ anti-spike antibody attached gold nanoparticles can break the lipid membrane of pseudo SARS-CoV-2.

It is now well documented that SARS-CoV-2 fusion with host cell membranes proceeds through interactions between spike proteins of the virus and the ACE2 receptor on the surface of human cells.^5–16,34–41^ To find out whether anti-spike antibody attached gold nanoparticles have the capability to block entry of the virus into cells, we used pseudo SARS-CoV-2 (# C1110G, Montana Molecular, Bozeman, MT) as the model virus.^36–38^ For our inhibition experiment we used HEK293T cells, which express ACE2, by which SARS-CoV-2 entry in human cells is determined.^36–38^ As shown in Fig. 4A–C, anti-spike antibody attached gold nanoparticles block viral replication and virus spread in HEK293T cells. Our data show that the inhibition efficiency was 100% for 100 ng mL^−1^ anti-spike antibody attached gold nanoparticles and 60% for 10 ng mL^−1^ anti-spike antibody attached GNPs. In contrast, our experimental data indicate that the inhibition efficiencies for PEG coated GNPs and 100 ng mL^−1^ antibody only were very low, less than 1%. Thus, for the design of anti-spike antibody GNPs we used 100 ng mL^−1^ antibody, since our data indicated 100% inhibition efficiency in the presence of 100 ng mL^−1^ anti-spike-antibody attached gold nanoparticles.

The inhibition is possibly due to antibody attached gold nanoparticles binding to pseudo SARS-CoV-2, thereby inhibiting the virus from binding to the cell receptor,^14,34–41^ and as a result, preventing the virus from infecting the targeted cells.^34–41^ But also, as shown in Fig. 4D, antibody attached GNPs have the capability to break the lipid membrane of pseudo SARS-CoV-2, so that the virus particle collapses.^34–41^ And, as we and others have reported before, gold nanoparticles suppress viral infection by selectively cleaving disulfide bonds, which blocks membrane fusion and viral entry to the host cell.^14,34,35,38–41^ We believe that all the above discussed mechanisms are responsible for the 100% inhibition efficiency observed using the anti-spike antibody attached gold nanoparticles.

## Conclusions

We have developed novel anti-spike antibody attached gold nanoparticles for rapid virus diagnosis and inhibition. Our reported data demonstrate that antibody attached GNPs can be used for the naked eye detection of specific COVID-19 viral antigen or of pseudo SARS-CoV-2 *via* a simple colorimetric change. Our experimental data show that anti-spike antibody attached gold nanoparticle-based sensing of COVID-19 viral antigen or virus is very fast: they can be detected within a 5 minute time period. Our results show that a naked eye assay allows the presence of antigen or virus to be determined even at a concentration of 1 ng mL^−1^ for COVID-19 antigen and 1000 virus particles per mL for pseudo SARS-CoV-2. We have also demonstrated that, using anti-spike antibody and 4-aminothiophenol attached gold nanoparticle-based SERS, COVID-19 viral antigen or virus at very low concentrations can be diagnosed within 5 minutes. Our reported data show that, using a portable Raman analyzer, our antibody and 4-aminothiophenol attached gold nanoparticle-based SERS probe has the capability to detect COVID-19 antigen, even at a concentration of 4 pg mL^−1^ and virus at a concentration of 18 virus particles per mL. Our reported finite-difference time-domain (FDTD) simulation data indicate a 4-orders-of-magnitude Raman enhancement occurs in “hot spot” positions when gold nanoparticles formed aggregates.

Using HEK293T cells, which express angiotensin-converting enzyme 2 (ACE2), by means of which SARS-CoV-2 gains entry into human cells, we demonstrated that anti-spike antibody attached gold nanoparticles have the capability to control viral infection. Our reported data show that the inhibition efficiency is 100% for anti-spike antibody attached gold nanoparticles. On the other hand, for GNPs alone the inhibition efficiency is only around 1% and for 100 ng mL^−1^ antibody the inhibition efficiency is less than 1%. That our reported data indicate 100% inhibition efficiency in the presence of anti-spike antibody attached gold nanoparticles may be due to the fact that antibody attached gold nanoparticles bind to pseudo SARS-CoV-2, thereby inhibiting the virus from binding to cell receptors. Our data show that antibody attached GNPs block viral replication and virus spread in HEK293T cells. They also destroy the virus lipid membrane.

## Conflicts of interest

There are no conflicts of interest to declare.

## Supplementary Material

NA-003-D0NA01007C-s001
