# Supplementary material for: The rapid diagnosis and effective inhibition of coronavirus using spike antibody attached gold nanoparticles
Source: Nanoscale Adv. 2021 Jan 18;3(6):1588–96. doi: 10.1039/d0na01007c (PMC8323809; doi:10.1039/d0na01007c)
Supplement: NA-003-D0NA01007C-s001 [file NA-003-D0NA01007C-s001.pdf]

Electronic Supplementary Material (ESI) for Nanoscale Advances.
This journal is © The Royal Society of Chemistry 2021

Supporting Information

Rapid Diagnosis and Effective Inhibition of Corona Virus Using Spike Antibody Attached Gold Nanoparticle

Avijit Pramanik[1], Ye Gao[1], Dipanwita Mitra[2], Martin G. McCandless[2], Lauren A Fassero[2], Shamily Patibandla[1], Kalein Gates[1], Ritesh Tandon[2], and Paresh Chandra Ray[1] *

[1]Department of Chemistry and Biochemistry, Jackson State University, Jackson, MS, 39217, USA; E-mail: paresh.c.ray@jsums.edu; Fax: +16019793674

[2]Department: Microbiology and Immunology, University of Mississippi Medical Center, Jackson, MS, 39216, USA

Methods

Gold chloride, citric acid, sodium borohydride, anti-spike antibody, COVID-19 antigen, and other chemicals were purchased from Sigma-Aldrich (St. Louis, MO, USA). We purchased the HEK293T cell line and HEK293T cell line from the American Type Culture Collection, which were grown according to an ATCC procedure**.** The SARS-CoV-2 spike protein pseudotyped baculovirus ((#C1110G, Montano Molecular, Bozeman, MT) was purchased from Montan0 Molecular, Bozeman, Montana 59715.

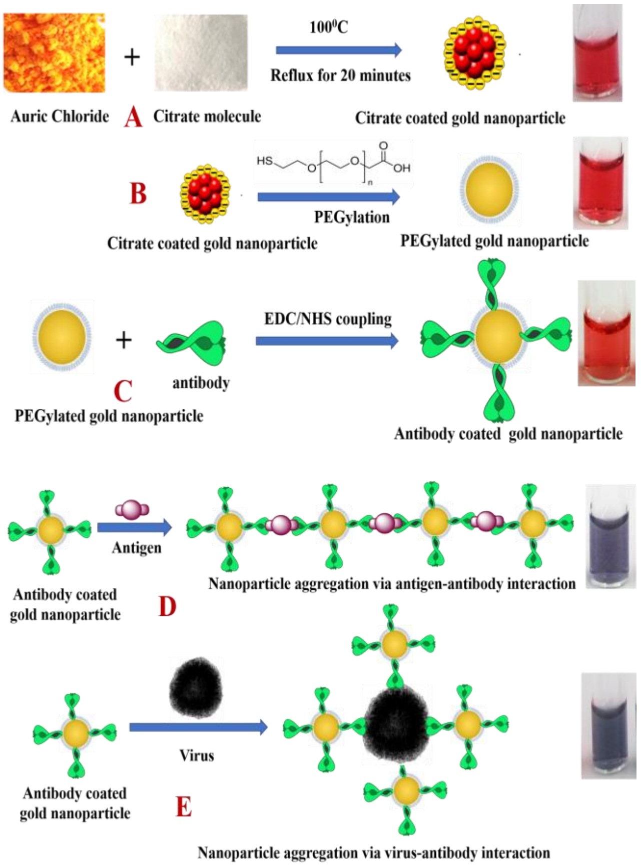

Figure S1: A) Scheme shows the synthetic path we have used for the development of citrate coated gold nanoparticle (inserted picture shows the color of citrate coated gold nanoparticle). B) Scheme shows the synthetic path we have used for the development of PEGylated gold nanoparticle (inserted picture shows the color of PEGylated gold nanoparticle). C) Scheme shows the synthetic path we have used for the development of anti-spike antibody attached gold nanoparticle (inserted picture shows the color of antibody attached gold nanoparticle). D) Scheme shows that in the presence of antigen, due to the antigen-antibody interaction gold nanoparticle aggregates (inserted picture shows the color of antibody attached gold nanoparticle in the presence of antigen). E) Scheme shows that in the presence of virus, due to the antigen-antibody interaction gold nanoparticle aggregates on the surface of virus (inserted picture shows the color of antibody attached gold nanoparticle in the presence of virus).

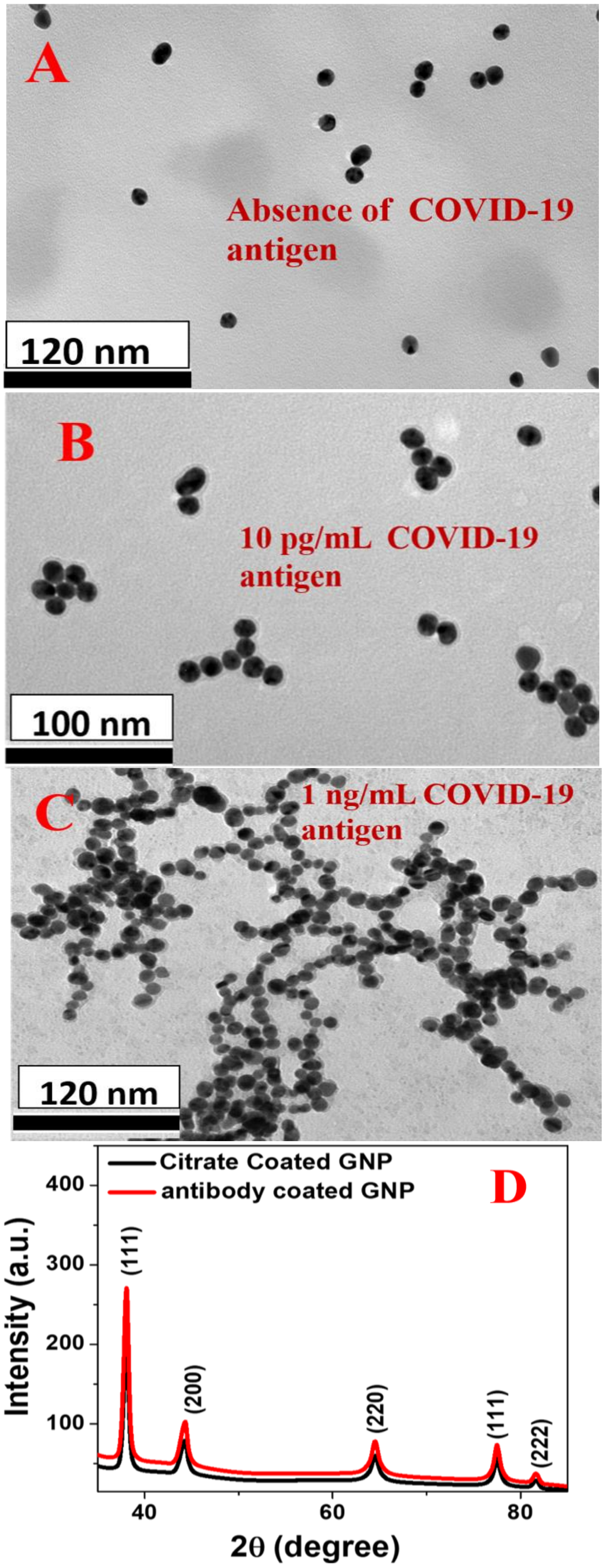

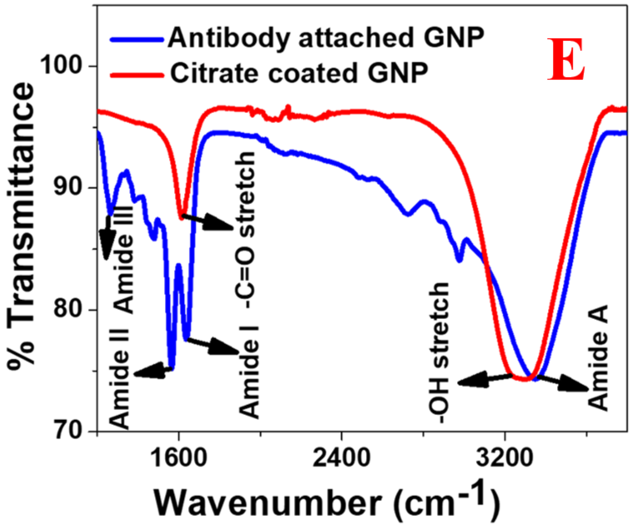

Figure S2: A) TEM shows the morphology of anti-spike antibody attached gold nanoparticle in the absence of COVID-19 antigen. B) TEM shows the morphology of antibody attached GNPs in the presence of 10 pg/mL of COVID-19 antigen (COVID-19 Spike Recombinant Antigen). C) TEM shows the morphology of antibody attached GNPs in the presence of 1 ng/mL of COVID-19 antigen. D) X-ray diffraction (XRD) spectra from citrate coated gold nanoparticle and antibody attached GNPs. E) FTIR spectra from citrate coated gold nanoparticle and antibody attached GNPs.

Development of 4-aminothiophenol and antibody conjugated GNP

As shown in Figure 3A, for rapid and highly sensitive identification via SERS, we have used 4-aminothiophenol as reporter molecules, which are attached to gold nanoparticle via Au-S bond. For this purpose, we have dissolved 4-amino-thiophenol in 5 mL water and then added it to antibody coated GNP using a syringe under vigorous stirring. After that the mixture was stirred at 60 °C for 3 h. Next the mixture was stabilized at room temperature for several hours. In the next step, the 4-aminothiophenol attached GNPs were separated from unbounded GNPs and unbound 4-aminothiophenol through centrifugation.
